# Poor Sperm Chromatin Condensation Is Associated with Cryopreservation-Induced DNA Fragmentation and Cell Death in Human Spermatozoa

**DOI:** 10.3390/jcm13144156

**Published:** 2024-07-16

**Authors:** Jade Hallam, Peter Burton, Katherine Sanders

**Affiliations:** 1 School of Human Sciences, The University of Western Australia, Crawley, WA 6009, Australia; jade.hallam@research.uwa.edu.au; 2 Concept Fertility Centre, Subiaco, WA 6008, Australia; peterburton@iinet.net.au

**Keywords:** sperm chromatin condensation, aniline blue, semen cryopreservation, sperm cryodamage, sperm cryotolerance, post-thaw sperm quality, sperm DNA fragmentation, sperm apoptosis

## Abstract

**Background/Objectives**: Semen cryopreservation is routinely performed in fertility clinics for a variety of reasons, including fertility preservation and storage of donor sperm, yet the freeze–thaw process leads to cellular damage via ice crystal formation, osmotic shock, and supraphysiological levels of oxidative stress. Sperm resistance to damage during the freeze–thaw process varies widely, yet the intrinsic factors associated with sperm cryotolerance are largely unknown. The study aimed to investigate whether poor chromatin condensation renders sperm vulnerable to DNA fragmentation and cell death induced by the freeze–thaw process. **Methods**: Participants (n = 51) from the general community who met the inclusion criteria collected a semen sample after 3–8 days of abstinence. Neat semen samples underwent traditional semen analysis, aniline blue (AB)-eosin staining for chromatin condensation, the terminal deoxynucleotidyl transferase dUTP nick end labelling (TUNEL) assay for DNA fragmentation, and the Annexin V assay for apoptosis/necrosis, prior to being cryopreserved using the liquid nitrogen vapour method and stored at −196 °C. Stored samples were later thawed at room temperature and processed using density gradient centrifugation. Motile sperm concentration, DNA fragmentation and apoptosis/necrosis were analysed in post-thaw samples. **Results**: As indicated by a significant interaction effect in linear mixed models, an increased proportion of AB-positive sperm in the pre-freeze sample exacerbated the adverse effect of freezing on sperm DNA fragmentation (*p* = 0.004), late apoptosis (*p* = 0.007), and necrosis (*p* = 0.007). AB-staining was positively correlated with all three parameters in the post-thaw sample (all r_s_ ≥ 0.424, all *p* < 0.01) and remained significant after adjusting for neat sperm concentration (all partial r_s_ ≥ 0.493, all *p* < 0.01). Similarly, AB-staining was significantly correlated with the percentage point change in sperm DNA fragmentation (r_s_ = 0.366, *p* = 0.014) and necrosis (r_s_ = 0.403, *p* = 0.009), both of which remained significant after adjusting for neat sperm concentration (both partial r_s_ ≥ 0.404, both *p* < 0.01), and borderline significantly correlated with percentage point change in late apoptosis (r_s_ = 0.307, *p* = 0.051). **Conclusions**: Sperm with poorly condensed chromatin may be more susceptible to cellular damage during the freeze–thaw process, independent of pre-freeze sperm concentration. These findings may help to explain the intrinsic variation in sperm resistance to cryodamage within and between individuals that is poorly understood.

## 1. Introduction

Semen cryopreservation is routinely performed in fertility clinics for a variety of reasons, including to preserve fertility prior to gonadotoxic cancer treatment, hormone therapies, or vasectomy, to store donor sperm, and to store sperm from men who may be unable to provide a fresh sample on the day of the ART procedure [1]. Despite its routine use, the freeze–thaw process exposes spermatozoa to intracellular and extracellular ice formation, osmotic stress, and oxidative stress [2]. Through these mechanisms, semen cryopreservation has a detrimental effect on various measures of sperm quality, including motility, morphology, and DNA fragmentation [3,4,5,6,7,8].

Sperm DNA fragmentation refers to single- and double-strand breaks in DNA. Sperm DNA fragmentation can be assessed using various direct and indirect methods [9]. Direct methods detect actual DNA strand breaks using probes or dyes, while indirect tests measure the susceptibility of the DNA to denaturation, which is characteristic of fragmented DNA [9,10]. Terminal deoxynucleotidyl transferase dUTP nick end labelling (TUNEL) is one such direct test of sperm DNA fragmentation that is frequently used in clinical practice [9]. While the standardisation and utility of DNA fragmentation assessment in clinical practice are still debated, and further research is needed [9,10], a considerable body of literature has demonstrated an association between increased sperm DNA fragmentation and poorer clinical outcomes following intrauterine insemination (IUI) [11], in vitro fertilisation (IVF) [12,13,14], and to a lesser degree, intracytoplasmic sperm injection (ICSI) [15,16,17]. Specifically, high sperm DNA fragmentation has been associated with impaired embryo development [18,19,20,21,22,23] and recurrent pregnancy loss [24,25,26,27] and is speculated to negatively affect the health of the offspring [28,29]. These outcomes are affected because sperm with fragmented DNA may still have the capacity to fertilise an oocyte, yet if the degree of fragmentation exceeds the DNA repair capacity of the oocyte, the damage may persist in the embryonic genome [28,30]. Therefore, reducing and preventing sperm DNA fragmentation prior to assisted reproductive technology is paramount.

The origin of sperm DNA fragmentation is related to the process of sperm development and the unique architecture of the mature spermatozoa. In the latter stages of normal spermiogenesis, the majority (approximately 85%) of histones, around which the sperm chromatin is wound, are replaced with protamines, allowing for chromatin compaction [31,32]. As sperm transit the epididymis, disulphide bonds form within and between protamines, allowing for further compaction and stabilisation of the chromatin [33]. The product is a spermatozoon with tightly condensed chromatin that is largely protected from oxidative attack and distinct from the looser chromatin structure of somatic cells [34,35]. The sperm cell structure is also unique in that it contains minimal cytoplasm and, therefore, depends on tightly packed chromatin and extracellular antioxidants for protection from oxidative damage [36,37].

The mechanism of sperm DNA fragmentation has been described as a ‘two-step’ process, a hypothesis proposed by De Iuliis et al. [35,38] and further discussed by Aitken and De Iuliis [39], involving oxidative stress as the primary driving factor. The first step of the ‘two-step’ hypothesis proposes that when the developmental process of chromatin condensation is impaired, the resultant spermatozoa have poorly condensed chromatin. The poorly condensed chromatin renders the spermatozoa vulnerable to oxidative attack and with a tendency to default to the apoptotic pathway [36,40,41]. The second step involves exposure of these vulnerable spermatozoa to excess reactive oxygen species (ROS) and/or inadequate antioxidant protection in the microenvironment [35,38]. Sperm exposure to oxidative stress appears to trigger the apoptotic pathway, characterised by the externalisation of phosphatidylserine to the outer leaflet of the cell membrane and further generation of ROS by mitochondria, leading to a self-propagating cascade of oxidative stress [40]. Ultimately, oxidative stress leads to the formation of 8-oxy-2′-deoxyguanosine (8-OHdG) adducts on the DNA, followed by DNA strand breaks [35] and, potentially, cell death [40]. These processes may occur both prior to ejaculation, as sperm transit and are stored in the epididymis, and post-ejaculation, such as during the preparation of the sperm for assisted reproductive technology [42,43]. In particular, semen cryopreservation exposes sperm to supraphysiological levels of oxidative stress and leads to increased apoptosis, necrosis, and DNA fragmentation [2,3,4,44,45,46].

While the mechanisms of sperm cryodamage are well studied, there is considerable variability in sperm resistance to cryodamage that is poorly understood, both between individuals and between samples from the same individual [47,48]. It has been reported that the response to freezing is associated with baseline semen parameters, as reduced cryorecovery has been found in samples with poorer pre-freeze motility and morphology [47,48,49,50]. A small number of studies have identified biomarkers in human semen that may predict the recovery of motile sperm after cryopreservation, including spermatozoa and seminal plasma fatty acid composition [51], corrected cholesterol [52], seminal plasma concentration of anti-mullerian hormone and inhibin B [53], and a number of proteins, such as enolase1 (ENO1), vimentin (VIM), tektin-1 (TEKT1) [54], aconitate (ACO2), and pyruvate kinase M2 (PKM2) [55]. Extensive research has also been conducted on markers of freezability of animal sperm; however, many appear to be species-specific [56]. Overall, the intrinsic factors affecting human sperm cryotolerance, particularly DNA damage and apoptosis during the freeze–thaw process, remain largely unknown [1].

It is widely stated that sperm with poor chromatin condensation are more susceptible to oxidative attack and subsequent DNA damage, as described in the ‘two-step’ hypothesis above [35,38]. Chromatin condensation can be measured using a variety of staining techniques, each with subtle differences in the specific defect detected [57]. One of the commonly used stains is aniline blue (AB), which is an acidic dye that binds to lysine-rich histones in the nucleus. As such, sperm nuclei with loose chromatin packaging take up the stain and appear dark blue when viewed under a brightfield microscope [57]. Several studies have reported that post-thaw samples have an increased proportion of AB-positive sperm, suggesting that the freeze–thaw process leads to chromatin decondensation [58,59,60,61]. However, the relationship between pre-freeze chromatin condensation and cryopreservation-induced DNA fragmentation and cell death has not, to our knowledge, been directly assessed.

Therefore, the present study aimed to investigate the relationship between pre-freeze sperm chromatin condensation, as measured with aniline blue staining, and cryopreservation-induced DNA fragmentation and cell death. A secondary aim was to investigate whether any observed effects are independent of baseline semen parameters. It was hypothesised that samples with poor sperm chromatin condensation are more susceptible to cryopreservation-induced DNA fragmentation and cell death.

## 2. Materials and Methods

### 2.1. Participant Recruitment, Inclusion, and Sample Collection

Male participants (n = 55) were recruited from the general community through advertisements throughout the University of Western Australia. Participants were included if they provided informed consent and fit the following inclusion criteria: BMI between 18.5 and 24.9, non-smoker, aged between 18 and 40 years, and negative for HIV, Hepatitis B, and Hepatitis C. Participants were randomly allocated to abstain from ejaculation for a period of 3–4, 5–6, or 7–8 days prior to sample collection as part of a larger study. Abstinence-related effects on sperm quality are not reported in the present study. Participants collected a semen sample by masturbation at Concept Fertility Centre (Subiaco, WA, USA); four participants were subsequently excluded due to missing a portion of the ejaculate, leaving a final sample size of 51 participants. Participants also completed a questionnaire with questions relating to cold and flu symptoms, medications, and supplements within the three months preceding sample collection, as well as any significant medical history. The study was approved by the UWA Human Research Ethics Committee (RA/4/20/5855, March 2020).

### 2.2. Semen Analysis and Cryopreservation

All semen samples were allowed to liquefy at room temperature for 30 min, at which time they underwent semen analysis. Semen analysis was conducted according to clinic protocol, which is adapted from the World Health Organization (WHO) Laboratory Manual for the Examination and Processing of Human Semen [62]. Briefly, a 10 µL aliquot of the semen sample was placed onto a Makler chamber and viewed at 10× objective under a light microscope; two counts of sperm concentration/motility were performed, and the results were averaged. For each count, at least 200 sperm were counted and classified as progressively motile, non-progressively motile, or immotile; these values were used to calculate the total sperm concentration (million/mL), total motility (%), and progressive motility (%).

Of the 51 neat samples collected and analysed, 48 were cryopreserved according to clinic protocol; three samples had insufficient volume for cryopreservation and were not frozen. An equal volume of Quinn’s Advantage^TM^ Sperm Freezing Medium (Origio, Måløv, Denmark) was added to semen in a drop-wise manner and thoroughly mixed. The semen–cryoprotectant mixture was immediately drawn up into 0.5ml straws and suspended in liquid nitrogen vapour approximately 10cm above the level of the liquid nitrogen for 30 min and then submerged and stored in liquid nitrogen (−196 °C). Several months later, straws were removed from liquid nitrogen, thawed at room temperature, and immediately processed via density gradient centrifugation; briefly, a discontinuous density gradient column was formed in a Falcon tube by underlaying 0.5 mL of 80% PureCeption^TM^ media underneath 0.5 mL of 40% PureCeption^TM^ media, maintaining a sharp interface between the two layers. One ml of the thawed semen–cryoprotectant mixture was overlayed onto the density gradient column and centrifuged at 310× *g* for 15 min. The pellet was resuspended in 2 mL of Quinn’s Advantage^TM^ Medium with HEPES containing 5% Human Serum Albumin and centrifuged at 520× *g* for 5 min. Finally, the supernatant was removed until a final volume of 0.5 mL remained, and the pellet was resuspended.

Pre-freeze (neat) semen samples were assessed for sperm chromatin condensation (the aniline blue-eosin staining method). Sperm DNA fragmentation (TUNEL) and apoptosis/necrosis (Annexin V assay) were assessed both before and after cryopreservation. The three methods of sperm quality are described in more detail below.

### 2.3. Sperm Chromatin Condensation (Aniline Blue-Eosin Staining)

Neat semen (10 µL) was smeared onto a positively charged microscope slide and allowed to air dry. On the same day, slides were fixed by submerging them in 10% formalin for 10 min and washed using water. The fixed slide was stained using 5% acidic aniline blue in 4% glacial acetic acid (pH of 3.5) for 5 min, washed in water, and counterstained with 0.5% aqueous eosin for one minute. Finally, the slide was washed in water, allowed to air dry and covered with a glass coverslip using UltraMount No. 4 (Hurst Scientific, Forrestdale, Australia). All slides were stored at room temperature for later analysis.

Slides were viewed under a 100× oil immersion objective on a brightfield microscope, and at least 200 spermatozoa were counted using a sampling grid. Spermatozoa were classified as AB-positive if all or part of the sperm head was stained dark blue, while spermatozoa were classified as AB-negative if the sperm head was stained pink or pale blue. The observer was blinded throughout, and the slides were assessed in a random order.

### 2.4. Sperm DNA Fragmentation (TUNEL)

An aliquot of the neat (10 µL) or frozen-thawed (10–40 µL) sperm samples was smeared onto a positively charged microscope slide and allowed to air dry; the volume of the frozen-thawed aliquot was adjusted to ensure a sufficient density of sperm on the microscope slide. Air-dried slides were stored at room temperature, away from light, for later analysis.

Slides were analysed using the ApopTag Peroxidase In Situ Apoptosis Detection kit (S7100; Sigma-Aldrich, St. Louis, MO, USA), which detects DNA strand breaks with the TUNEL method. Prior to the assay, air-dried slides were fixed in a 3:1 solution of methanol and acetic acid for 5 min, washed in phosphate-buffered saline (PBS) for 5 min, permeabilised in 0.1% Triton-X 100 (in PBS) for 5 min, and again washed in PBS for 5 min. The assay was subsequently performed according to manufacturer instructions. Stained slides were covered with a glass coverslip using UltraMount No. 4 (Hurst Scientific, Australia) and stored at room temperature, away from light, for later analysis.

Slides were viewed under a 100× oil immersion objective on a brightfield microscope; sperm with partial or complete brown staining were considered TUNEL-positive (i.e., sperm with fragmented DNA), and blue-stained sperm were considered TUNEL-negative (i.e., sperm without fragmented DNA). A minimum of 400 sperm were counted per slide using a sampling grid, and the final DNA fragmentation index (DFI) was calculated as the number of TUNEL-positive sperm divided by the total number of sperm counted as a percentage. DFI was not calculated for three frozen-thawed samples, as fewer than 400 sperm could be counted; a total of 51 neat and 45 frozen-thawed samples were assessed for DNA fragmentation.

### 2.5. Sperm Apoptosis/Necrosis (Annexin V Assay)

The annexin V assay (Millipore, Burlington, MA, USA) was conducted on neat and post-thaw samples on the day of sample collection or thawing, respectively, according to the manufacturer’s instructions and as described by our laboratory previously [63]. Samples were diluted to a sperm concentration of 0.1–0.5 million/mL in Quinn’s advantage medium with HEPES (Origio, Denmark) containing 5% Human Serum Albumin. Diluted samples were mixed with an equal volume of the Annexin-V reagent, which contains both Annexin-V and 7-aminoactinomycin (7-AAD), to a total volume of 200 µL and incubated away from light for 20 min. Following incubation, samples were pipette-mixed and immediately analysed using the Muse Cell Analyzer (Millipore, USA), a benchtop flow cytometer with the minimum number of events set as 3000 events per run.

The Annexin-V reagent includes both Annexin-V (Ann), which binds to phosphatidylserine that has translocated to the outside of the cell, and 7-AAD, which is a membrane-impermeant DNA binding that discriminates live and dead cells. Using flow cytometry and previously determined fluorescent thresholds, cells were classified as live (Ann −ve/7-AAD −ve), early apoptotic (Ann +ve/7-AAD −ve), late apoptotic or dead (Ann +ve/7-AAD +ve), or necrotic (Ann −ve/7-AAD +ve). Gate thresholds were initially set using a normozoospermic sample that had been divided into two aliquots: one aliquot was repeatedly dunked in liquid nitrogen and used as a positive control, while the second aliquot was processed using density gradient centrifugation and used as a negative control. Eight consecutive neat samples did not undergo the Annexin V assay due to equipment malfunction; a total of 43 neat and 48 frozen-thawed samples were assessed using the Annexin V assay.

### 2.6. Statistical Analysis

All statistical analyses were conducted using R (version 4.3.3). Summary statistics are reported as median (IQR). As the proportion of AB-positive sperm did not follow a normal distribution, Spearman’s rank-order correlation coefficient (r_s_) was reported for all bivariate correlations and, where appropriate, partial Spearman’s correlation was used for adjusted analyses. Linear mixed models were used to test for an interaction effect between the proportion of AB-positive sperm and freeze-status on DFI (%), early apoptosis (%), late apoptosis (%), and necrosis (%), including participant ID as a random effect. All models were checked for heteroscedasticity and distribution of residuals; dependent variables were transformed, if needed, to meet the assumption of normality. Where a significant interaction was observed, Spearman’s correlation was performed within pre-freeze and post-thaw subgroups. Finally, in the subset of participants with both pre-freeze and post-thaw values for the given outcome measure, the post-cryopreservation change in DFI (n = 45), early apoptosis, late apoptosis, and necrosis (n = 41) was calculated as the post-thaw value minus the pre-freeze value and reported as a change in percentage points (p.p.). A *p*-value of <0.05 was considered statistically significant.

## 3. Results

The median (IQR) ejaculatory abstinence period was 5 (3) days. The median age (IQR) of participants was 21.33 (4.42) years. Of the 51 participants, 20 reported cold and flu symptoms (e.g., headache, sore throat, fever) in the three months preceding sample collection; seven of those tested positive for COVID-19 between three months and up to two weeks prior to sample collection. The seven participants who had recently tested positive for COVID-19 had a proportion of AB-positive sperm (%) that was similar to that of the remaining 44 participants, with median (IQR) values of 9.90% (5.73) and 9.48% (12.60), respectively. Three participants reported a history of testicular injury, two of which reported a history of testicular torsion; the proportions of AB-positive sperm for these three participants were 3.90%, 18.10%, and 28.22%. Only one participant reported consuming 14+ standard units of alcohol per week, with the proportion of AB-positive sperm in their sample at 27.19%. Where possible, individual data points corresponding to participants who reported recent COVID, a history of testicular injury, or regularly consuming 14+ standard units of alcohol per week have been indicated in figures accordingly. The overall median (IQR) proportion of AB-positive sperm was 9.71% (11.70).

### 3.1. Correlation between the Proportion of AB-Positive Sperm and Conventional Semen Parameters

The median (IQR) of conventional semen parameters, and their Spearman’s correlation coefficient with the proportion of AB-positive sperm, is summarised in Table 1. The proportion of AB-positive sperm was significantly negatively correlated with sperm concentration yet was independent of all other semen parameters.

### 3.2. Association between the Proportion of AB-Positive Sperm and Progressively Motile Sperm in Pre-Freeze vs. Post-Thaw Samples

In a linear mixed model, there was a significant interaction effect between the proportion of AB-positive sperm (%) and the freeze status of the sample (pre-freeze or post-thaw) on the progressively motile sperm concentration (*p* = 0.004). The progressively motile sperm concentration was 47.5 (34.0) million/mL in the pre-freeze sample and was negatively correlated with the proportion of AB-positive sperm (r_s_ = −0.426, *p* = 0.002, n = 51). The progressively motile sperm concentration was 2.5 (4.4) million/mL following cryopreservation, thawing, and density gradient processing and was also negatively correlated with the pre-freeze proportion of AB-positive sperm (r_s_ = −0.434, *p* = 0.003, n = 45). The recovery of progressively motile sperm following cryopreservation, thawing, and density gradient processing (post-thaw concentration/neat concentration) was negatively correlated with the pre-freeze proportion of AB-positive sperm (r_s_ = −0.368, *p* = 0.013, n = 45).

While the proportion of AB-positive sperm was not correlated with pre-freeze progressive motility (%; Table 1), it was negatively correlated with post-thaw progressive motility (r_s_ = −0.424, *p* = 0.004, n = 45), which had a median (IQR) of 59.6% (28.5).

### 3.3. Association between the Proportion of AB-Positive Sperm and Measures of DNA Fragmentation and Cell Death in Pre-Freeze vs. Post-Thaw Samples

**Figure 1:**
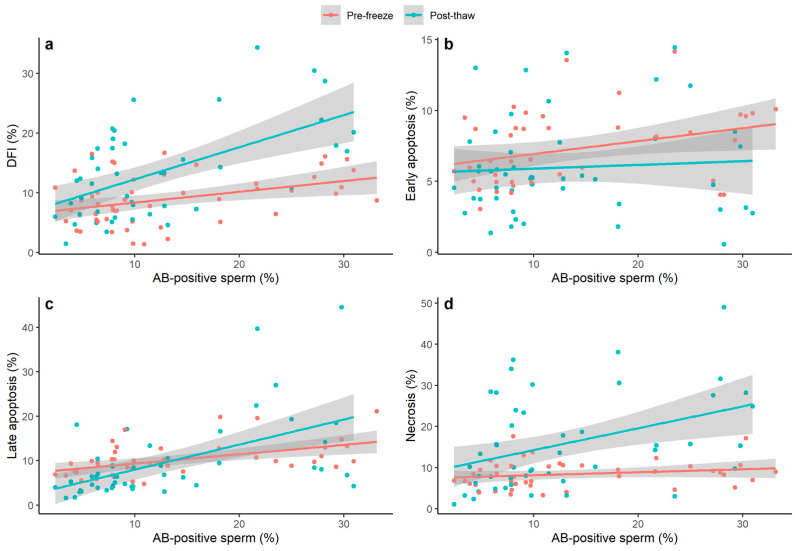
The association between the percentage of pre-freeze AB-positive sperm and the following outcome variables before and after cryopreservation: (**a**) DFI (percentage of TUNEL-positive sperm); (**b**) Early apoptosis (percentage of Ann+/7-AAD− sperm); (**c**) Late apoptosis (percentage of Ann+/7-AAD+ sperm); (**d**) Necrosis (percentage of Ann−/7-AAD+ sperm). Data shown are raw values with linear trend lines (±95% CI) per group. DFI was measured pre- and post-cryopreservation in 51 and 45 samples, respectively, while apoptosis/necrosis outcomes were measured pre- and post-cryopreservation in 43 and 48 samples, respectively. By using linear mixed models, a significant interaction was observed between AB-positive sperm and the freeze status (pre-freeze/post-thaw) of the sample for DNA fragmentation (*p* = 0.004), late apoptosis (*p* = 0.007), and necrosis (*p* = 0.007). No significant interaction was observed in the model of early apoptosis. Late apoptosis (%) was log-transformed, and necrosis (%) was square root-transformed prior to analysis. AB = aniline blue; DFI = DNA fragmentation index; Ann = Annexin V; 7-AAD = 7-aminoactinomycin.

Linear mixed models were used to test for a possible interaction effect between the proportion of AB-positive sperm (%) and the freeze status of the sample (pre-freeze or post-thaw) on the proportion of DNA fragmented, early apoptotic, late apoptotic, and necrotic sperm (%); a significant interaction effect was observed for the proportion of DNA fragmented, late apoptotic, and necrotic sperm (Figure 1).

Where a significant interaction effect was observed in the linear mixed model, separate Spearman’s correlations were performed for the relationship between the proportion of AB-positive sperm and the outcome variable in pre-freeze and post-thaw samples (Table 2). In the pre-freeze sample, the proportion of AB-positive sperm was significantly positively correlated with that of DNA-fragmented and late apoptotic sperm, while there was no significant relationship with the proportion of necrotic sperm. The proportion of AB-positive sperm was positively correlated with all three outcome variables in the post-thaw samples, and the strength of the correlations was stronger than prior to cryopreservation. All post-thaw correlations persisted after adjustment for initial neat sperm concentration (Table 2).

### 3.4. Correlation between the Proportion of AB-Positive Sperm in the Neat Ejaculate and the Percentage Point Change in Measures of DNA Fragmentation and Cell Death following the Freeze–Thaw Process

**Figure 2:**
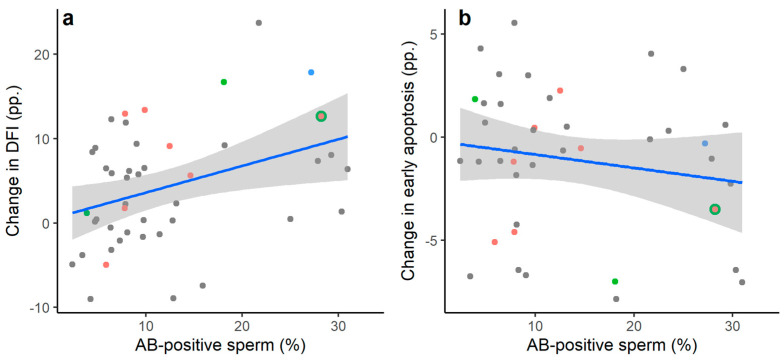

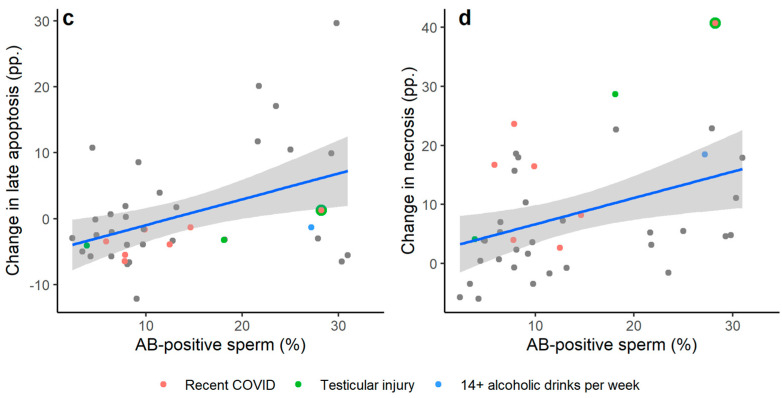
The association between the percentage of pre-freeze AB-positive sperm in the neat ejaculate and the percentage point change in the following outcome variables following the freeze–thaw process: (**a**) DNA fragmentation index (DFI; percentage of TUNEL-positive sperm); (**b**) Early apoptosis (percentage of Ann+/7-ADD− sperm); (**c**) Late apoptosis (percentage of Ann+/7-ADD+ sperm); (**d**) Necrosis (percentage of Ann−/7-ADD+ sperm). Data shown are raw values with linear trend lines (±95% CI). Significant Spearman’s rank-order correlations were observed between the proportion of AB-positive sperm in the neat ejaculate and the percentage point change in DFI (r_s_ = 0.366, *p* = 0.014) and necrosis (r_s_ = 0.403, *p* = 0.009). A non-significant correlation was observed for the percentage point change in late apoptosis (r_s_ = 0.307, *p* = 0.051), and no significant correlation was observed for early apoptosis (r_s_ = −0.133, *p* = 0.408). Coloured data points represent individuals who reported testing positive for COVID-19 within three months prior to sample collection, a history of testicular injury, or regularly consuming 14+ alcoholic drinks per week. N = 45 for DFI; n = 41 for measures of apoptosis and necrosis.

Using Spearman’s correlation, the proportion of AB-positive sperm (%) was significantly positively associated with the percentage point change in DNA-fragmented sperm and necrotic sperm induced by the freeze–thaw process (Figure 2), and these remained after adjustment for neat total sperm concentration (DNA-fragmented sperm partial r_s_ = 0.404, *p* = 0.005, n = 45; necrotic sperm partial r_s_ = 0.487, *p* = 0.002, n = 41). No significant correlation was observed between the proportion of AB-positive sperm and the percentage point change in late apoptotic sperm or early apoptotic sperm (Figure 2).

## 4. Discussion

While it is well established that cryopreservation is damaging to sperm quality and that there is considerable inter- and intra-individual variability in the resistance of sperm to cryodamage, factors affecting this variability are poorly understood [1]. In the present study, semen samples with poor sperm chromatin condensation, indicated by a relatively high proportion of AB-positive sperm, were more susceptible to cryopreservation-induced sperm DNA fragmentation, late apoptosis, and necrosis. Furthermore, AB-staining in the pre-freeze sample was associated with reduced recovery of progressively motile sperm after thawing and density gradient processing. Additionally, AB-staining was independent of most semen parameters in the neat sample, bar a negative correlation with sperm concentration. While it is widely stated that sperm chromatin protamination and condensation are protective against oxidative assault [35,38,64,65,66,67], the present study is the first, to our knowledge, to directly assess the relationship between pre-freeze chromatin condensation and vulnerability to cryopreservation-induced damage in human sperm.

It has long been accepted that the highly compacted structure of chromatin in mature spermatozoa serves to protect the paternal genome from external damage during transit through the male and female reproductive tract [67]. During the latter stages of spermatogenesis, approximately 85% of histones in the sperm nucleus are replaced by transition proteins, which are in turn replaced by protamines, resulting in DNA that is packaged into doughnut-shaped toroids [31,32]. The nucleoprotamine complexes are further compacted and stabilised by disulphide bonds that form within and between protamines during epididymal transit [33]. As described in the ‘two-step’ hypothesis put forward by De Iuliis et al. [35], any impairment of this process leads to sperm with poorly compacted chromatin that is vulnerable to oxidative attack and subsequent DNA strand breaks. Additionally, spermatozoa with poor chromatin remodelling are thought to default to the apoptotic pathway, which in turn triggers further ROS generation in a self-perpetuating cycle [40,68,69].

The protective role of chromatin condensation likely explains the finding of the present study that samples with poor chromatin condensation, as measured with aniline blue staining, are more susceptible to DNA fragmentation and cell death during the freeze–thaw process. While it is well established that cryopreservation exposes sperm to supraphysiological ROS levels and impacts sperm DNA integrity, a novel finding of this study is that samples with poor chromatin condensation incur relatively more damage during the freeze–thaw process. The present study demonstrates that samples with adequate levels of chromatin condensation largely maintain their DNA integrity following exposure to cryopreservation, while those with relatively poorer chromatin condensation are more likely to suffer DNA strand breaks and cell death during the freeze–thaw process, even if the pre-freeze levels of DNA fragmentation were comparable. This may have implications for the clinical utility of DNA fragmentation testing, which is typically performed using neat semen in instances where cryopreserved sperm will be used for IUI, IVF, or ICSI. Furthermore, aniline blue staining alongside DNA fragmentation assessment may provide insight into the likely DNA integrity of the post-thaw sample; however, further studies are required.

While aniline blue staining has not previously been explored as a predictor of sperm cryorecovery, other studies have found that cryorecovery is associated with baseline semen parameters such as motility and morphology [47,48,50,67]. Additionally, men with underlying malignancies, such as men cryopreserving a sample prior to cancer treatment, have poorer post-thaw survival compared to donors [70]. The present study included participants who were recruited from the public, and the samples had primarily normal semen parameters. Of the semen parameters tested in the present study, aniline blue was correlated only with sperm concentration. However, many of the key correlations between aniline blue and cryopreservation-induced changes in sperm quality persisted after adjusting for neat sperm concentration. Therefore, the role of chromatin condensation in sperm susceptibility to cryopreservation-induced damage is likely independent of baseline semen parameters; it should be noted, however, that morphology was not assessed in this study. It is also worth noting that the median abstinence length of 5 days, owing to the randomly allocated abstinence periods between 3 and 8 days of the larger study, is in the upper range of typically recommended abstinence periods of between 2 and 5 days [71,72]. Further studies are needed to extrapolate the findings to a clinical population with ejaculatory abstinence periods closer to recommended norms.

Beyond baseline semen parameters, other studies have considered a number of potential biomarkers of cryotolerance, including spermatozoa and seminal plasma fatty acid composition [51], corrected cholesterol [52], seminal plasma concentration of anti-mullerian hormone and inhibin B [53], and a number of proteins, such as enolase1 (ENO1), vimentin (VIM), tektin-1 (TEKT1) [54], aconitate (ACO2) and pyruvate kinase M2 (PKM2) [55]. In the present study, sperm chromatin condensation was moderately correlated with post-thaw DNA fragmentation and cell death, accounting for 17–30% of the variance. As such, it is likely that other factors relating to sperm chromatin, cellular structure, or seminal plasma composition are also involved, which is to be expected given the complex and multifactorial nature of freeze–thaw damage and sperm quality. Future research might aim to compare the utility of AB-staining to other proposed biomarkers, as well as any interplay between AB-staining and other factors, in terms of predicting cryopreservation outcomes.

While the present study has demonstrated a relationship between sperm chromatin condensation and the level of DNA fragmentation and cell death induced by semen cryopreservation, it is worth noting that newer and optimised sperm freezing protocols may reduce the overall damage and dampen the observed relationship. For example, vitrification has been proposed as an alternative to conventional freezing methods, such as the liquid nitrogen vapour method used in the present study [73]. As the mechanism of cellular damage during vitrification differs from that of conventional methods, the findings of the present study should not be generalised to all freezing methods. Additionally, thawing straws at 37 °C, rather than room temperature, has been associated with favourable post-thaw survival [74,75]. The present study used density gradient centrifugation to process post-thaw samples, which has been shown to generate further ROS in some samples [43]. Alternative processing methods that are gentler on sperm, such as the ‘swim-up’ technique, could alter the observed effects. Finally, density gradient processing for removing seminal plasma, debris, and abnormal sperm prior to semen cryopreservation, rather than after thawing, has been suggested to improve post-thaw outcomes [76]. Samples with a high proportion of AB-positive sperm may benefit from this method, as sperm with poor chromatin condensation would likely be removed from the sample prior to the oxidative assault of the freeze–thaw process. However, further studies are needed to compare processing before cryopreservation to processing after cryopreservation in samples with a low or high proportion of AB-positive sperm.

## 5. Conclusions

In conclusion, the present study observed that poor sperm chromatin condensation, as measured using aniline blue staining in neat semen, predisposed sperm to increased DNA fragmentation, late apoptosis and necrosis following semen cryopreservation, thawing, and density gradient processing. While the damaging effects of cryopreservation are known, not all semen samples are affected equally; some samples are largely resistant to freeze–thaw damage, while others incur significant damage. The present study is, to our knowledge, the first to observe that sperm chromatin condensation is associated with the extent of sperm DNA fragmentation and cell death induced by the freeze–thaw process, independent of baseline semen quality. Thus, chromatin condensation plays a role in the intrinsic resistance or susceptibility of sperm to freeze–thaw damage. The findings help to explain the considerable inter- and intra-individual variation observed in sperm cryotolerance that is often observed in the clinical laboratory yet is poorly understood. Future research should assess the role that chromatin condensation plays in cryorecovery in a subfertile patient population and explore potential clinical implications, such as the utility of pre-freeze DNA fragmentation assessment for the use of cryopreserved sperm and the potential for targeted modification of freezing protocols.

## Figures and Tables

**Table 1:** Median (IQR) of conventional semen parameters and Spearman’s rank-order correlation coefficient (r_s_) with the proportion of AB-positive sperm (n = 51). IQR = Interquartile range.

	Median (IQR)	R_s_	*p*
Semen volume (mL)	4.14 (3.06)	0.165	0.247
Sperm concentration (million/mL)	83.17 (62.7)	−0.381	0.006 **
Total sperm count (million)	342.78 (272.65)	−0.162	0.256
Total motility (%)	65.60 (17.87)	−0.175	0.220
Progressive motility (%)	59.48 (16.12)	−0.223	0.115

** *p* < 0.01.

**Table 2:** Spearman’s correlation (R_s_) between the proportion of AB-positive sperm in the neat ejaculate and the proportion of DNA-fragmented late apoptotic and necrotic sperm in the samples pre- and post-cryopreservation, thawing, and density gradient processing. Correlations are shown both unadjusted and adjusted (using Partial Spearman’s) for neat sperm concentration. AB = aniline blue; DFI = DNA fragmentation index; Ann = Annexin V; 7-AAD = 7-aminoactinomycin.

		Pre-Freeze		Post-Thaw	
		Unadjusted	Adjusted	Unadjusted	Adjusted
DFI (TUNEL+)		R_s_ = 0.327 *p* = 0.019 *	Partial R_s_ = 0.261 *p* = 0.055	R_s_ = 0.492 *p* < 0.001 ***	Partial R_s_ = 0.513 *p* < 0.001 ***
		N = 51		N = 45	
Late apoptosis (Ann+/7-AAD+)		R_s_ = 0.520 *p* < 0.001 ***	Partial R_s_ = 0.531 *p* < 0.001 ***	R_s_ = 0.555 *p* < 0.001 ***	Partial R_s_ = 0.527 *p* < 0.001 ***
		N = 43		N = 48	
Necrosis (Ann−/7-AAD+)		R_s_ = 0.248 *p* = 0.109	Partial R_s_ = 0.212 *p* = 0.115	R_s_ = 0.424 *p* = 0.003 **	Partial R_s_ = 0.493 *p* = 0.001 **
		N = 43		N = 48	

* *p* < 0.05, ** *p* < 0.01, *** *p* < 0.001.

## Data Availability

The datasets presented in this article are not available because the data are part of an ongoing study and are subject to restriction for ethical reasons. Requests to access the datasets should be directed to Dr. Katherine Sanders (kathy.sanders@uwa.edu.au).
